# Natural language processing of radiology reports for the detection of thromboembolic diseases and clinically relevant incidental findings

**DOI:** 10.1186/1471-2105-15-266

**Published:** 2014-08-07

**Authors:** Anne-Dominique Pham, Aurélie Névéol, Thomas Lavergne, Daisuke Yasunaga, Olivier Clément, Guy Meyer, Rémy Morello, Anita Burgun

**Affiliations:** Department of Biostatistics and Clinical Research, CHU de Caen, Caen, F-14000 France; Biomedical Informatics and Public Health Department, University Hospital HEGP, AP-HP, Paris, France; LIMSI-CNRS, rue John von Neumann, Orsay, F-91043 France; Department of Radiology, CHU de Caen, Caen, F-14000 France; Radiology department, Assistance Publique- Hôpitaux de Paris, Hôpital Européen Georges-Pompidou, 20, rue Leblanc, Paris, 75015 France; Université Paris-Descartes, INSERM UMR-S970 Paris Cardiovascuar Research center – PARCC, Paris, France; Pneumology department, Assistance Publique- Hôpitaux de Paris, Hôpital Européen Georges-Pompidou, 20, rue Leblanc, Paris, 75015 France; INSERM UMR_S 872 Team 22: Information Sciences to support Personalized Medicine, Sorbonne Paris Cité, Faculté de Médecine, Université Paris Descartes, Paris, France

**Keywords:** Natural language processing, Medical informatics, Embolism and thrombosis/diagnosis, Phlebography, Incidental findings, Human

## Abstract

**Background:**

Natural Language Processing (NLP) has been shown effective to analyze the content of radiology reports and identify diagnosis or patient characteristics. We evaluate the combination of NLP and machine learning to detect thromboembolic disease diagnosis and incidental clinically relevant findings from angiography and venography reports written in French. We model thromboembolic diagnosis and incidental findings as a set of concepts, modalities and relations between concepts that can be used as features by a supervised machine learning algorithm. A corpus of 573 radiology reports was de-identified and manually annotated with the support of NLP tools by a physician for relevant concepts, modalities and relations. A machine learning classifier was trained on the dataset interpreted by a physician for diagnosis of deep-vein thrombosis, pulmonary embolism and clinically relevant incidental findings. Decision models accounted for the imbalanced nature of the data and exploited the structure of the reports.

**Results:**

The best model achieved an F measure of 0.98 for pulmonary embolism identification, 1.00 for deep vein thrombosis, and 0.80 for incidental clinically relevant findings. The use of concepts, modalities and relations improved performances in all cases.

**Conclusions:**

This study demonstrates the benefits of developing an automated method to identify medical concepts, modality and relations from radiology reports in French. An end-to-end automatic system for annotation and classification which could be applied to other radiology reports databases would be valuable for epidemiological surveillance, performance monitoring, and accreditation in French hospitals.

## Background

### Thromboembolic disease diagnosis

The role of computed tomography (CT) in diagnosis has been growing as a result of advances in technology imaging including the recent emergence of multidetector and dual-energy CT. Pulmonary CT angiography (CTA) has become the first-line diagnostic imaging modality in most patients with suspected pulmonary embolism (PE). Similarly, CT venography (CTV) which concurrently follows CTA has also rapidly been adopted in the workup of PE for diagnosing deep vein thrombosis (DVT) [1]. Following the introduction of helical and multi-slice pulmonary CTA, many research studies were conducted to assess the contribution of CTV and reached different conclusions [2]. In a first group of studies, CTA-CTV improved the diagnostic yield in comparison with CTA alone [3–5]. In a second group [6, 7], the added yield of CTV was found too marginal to justify the additional irradiation. Furthermore, some of these studies suggested that CTV should be considered for patients at higher risk of venous thromboembolism (intensive care unit, history of malignancy, cardiovascular disease, recent surgery) or for patients with a high clinical probability of PE [5, 6] as well as in postpartum patients with suspected PE [8]. Overall, the role of CTV is less established than that of pulmonary CTA and questions remain about the utility of CTV.

The Hôpital Européen Georges Pompidou (HEGP) is a national reference center for vascular diseases. Patients with acute PE and who do not require fibrinolysis are directly oriented to the center. At HEGP, CTA is the first-line examination for patients suspected of having PE (except for patients with contrast medium injection contraindication) and indirect CTV is often performed following CTA [9]. As a result, the HEGP clinical data warehouse contains a large corpus of patient records with both CTA and CTV reports. This corpus is a valuable resource to analyze the contribution of each method in the diagnosis.

### Clinically relevant incidental findings

The increasing availability of imaging technologies has led to a growing number of *incidental findings*. Incidental findings are asymptomatic lesions that are discovered through routine radiographs (e.g., CT examination) obtained for other reasons [10–12]. For instance, a lung nodule in a patient with no history of lung cancer examined for suspected PE is considered an incidental finding. We focused our attention on the incidental clinically relevant findings that require clinical or radiologic follow-up, also called “incidentaloma”. These include: a nodule or mass located in an organ, including the lungs, liver, spleen, kidneys, bone, thyroid gland, pancreas, adrenal glands, and stomach; any lymph node > 1 cm and not associated with an infiltrate, or any lymph node > 3 cm, or multiple lymph nodes [10, 12]. In a study conducted at an emergency department [10], the estimated prevalence of these incidentalomas in chest CTA ordered to diagnose PE was 24% (141/589) while the prevalence of PE was only 9% (55/589). The evaluation of such findings from radiology reports places a huge burden on the health care system. The absence of an automated system to identify and track incidental findings is an important barrier to ensuring timely follow-up of patients especially with incidental findings on imaging examinations. In this study we designed an automated method to identify these medical findings related to thromboembolic disease and incidental findings from free-text radiology reports written in French using natural language processing methods, and evaluated the system on the HEGP corpus.

### Natural language processing for clinical free-text

The answers to important questions regarding thromboembolic disease diagnosis and incidental findings can be found in radiology reports. These are huge sets of free-text documents: for example HEGP radiologists produce about 66,000 reports each year. NLP has the potential to provide the means to analyze large quantities of documents rapidly and accurately, including clinical text [13]. Clinical narratives written in English have received a lot of attention from the NLP community in the past decades through the development of dedicated tools (e.g. MedLEE (Medical Language Extraction and Encoding System) [14, 15], cTAKES [16]) or repurposing of tools initially meant for processing the literature (e.g. MetaMap [17, 18]). Biomedical texts in languages other than English have received considerably less attention. French NLP teams regularly participate in the annual i2b2 challenges. Some of their work initially designed for the English language, was successfully transferred to French. Tools for automatic translation of terms from English to French have been developed [19–21]. Zweigenbaum *et al*. [22] worked on a project to pool lexical resources scattered among several sources in a unified medical lexicon for French (UMLF). Namer [23] described a method which enables neoclassical compound nouns and adjectives of a biomedical specialized corpus to be automatically related by synonymy, hyponymy and approximation links. Grabar *et al*. [24] developed a method for automatically acquiring synonymy resources. Deléger *et al*. [25] developed a medication extraction system for French clinical texts. Grouin *et al*. were able to compute the CHA2DS2-VASc score, which predicts the thromboembolic risk for patients with atrial fibrillation, using the automatically extracted medical concepts [26].

The purpose of the study presented herein was to analyze CT reports to automatically determine thromboembolic diagnosis and the technique used to reach it, as well as automatically identify clinically relevant incidental findings. We had the following specific aims: 1/set up a machine-learning based framework for automatic report analysis 2/assess the benefit of using NLP to extract clinically relevant concepts, modalities and relations between concepts 3/produce valuable resources to develop NLP tools able to automatically extract concepts, modalities and relations.

## Methods

In this section we describe the corpus, natural language processing tools and machine learning methods used in our work.

This study was approved by the institutional review board at HEGP which waived the need for informed consent (CDW_2013_0007 study from IRB #00001072).

### Corpus selection and pre-processing

The corpus selection was performed in two steps. In the first step, our objective was to extract the features of the reports associated with CTA/CTV in the context of PE. We queried the HEGP i2b2 clinical data warehouse using the label of the imaging procedures stored in the i2b2 observation blob field corresponding to the CT of vascular system performed in the year 2011. We obtained a set of 6,758 radiology exam reports. Among them, many documents reported on procedures corresponding to anatomical sites that were not of interest for our study (e.g., CTs labeled “CT of the vessels” that would explore the heart and the aortic root). In the second step, we drew a list of 8 key terms for CTV (e.g., “angioscanner thoracique”, “angioscanner du thorax”) and 6 for CTA (e.g., “phléboscan”, “phlebo-scan”). We used these lists of terms to select the CTA/CTV prescribed for PE. Specifically, we built a refined query that selected reports containing at least one term from each list. Among the 6,758 radiology exams retrieved by the initial query, only 573 were selected by the new query. We will use the term “corpus” to refer to that set of 573 reports.

We manually reviewed 200 randomly selected reports from the 6,758 initially retrieved to assess the performance of the refined query. We found that 78 reports were selected by the refined query. All corresponded to CTA and CTV prescribed in the context of PE (true positives). Among the remaining 122 reports, 70 corresponded to another indication or exam (true negatives) and 52 corresponded to relevant reports not selected by the query (false negatives). Overall, the query detected relevant reports with a precision of 100%, recall of 61% and F-measure of 68%. Further error analysis revealed that the query omitted to select reports that did not include the name of the exams performed, reports that used a different spelling from our list of keywords (mostly spelling errors and abbreviations), or reports that exhibited technical errors in the text conversion from the original record format.

The corpus was automatically de-identified using a ruled-based de-identification tool called MEDINA (MEDical INformation Anonymization) [27]. The system replaced patient and physician names by surrogate data and shifted dates within the report by a uniform random number of days so as to preserve the interval between dates. The results of the automatic de-identification process were validated by a physician (ADP). Then, a simple sentence segmenter (which identified sentence boundaries) and a tokenizer (which broke a stream of text into a sequence of tokens which roughly corresponds to ‘words’) were applied to our corpus. The corpus comprised a total of 33,344 tokens (7,407 unique). Report average length was 318 tokens. Although our radiology reports were in free-text format, the content was structured into five sections to describe: patient identification, indication of exam along with clinical information, exam protocol details, results, and conclusion. Each report displayed a section title followed by the corresponding free-text contents. We used a rule-based algorithm relying on regular expressions to split the reports into sections.

### Analysis of the radiology reports and design of an annotation scheme

The corpus of reports was manually reviewed by a physician (ADP) to label each report as: positive (or negative) CTA for presence (or absence) of PE, positive (or negative) CTV for presence (or absence) of DVT, and presence (or absence) of an incidental clinically relevant finding. This labeled data set was used as a gold standard for further machine learning classification evaluation (Table 1).

**Table 1:** **Distribution of thromboembolic diagnosis and incidental finding in the 573 radiology reports**

Diagnoses	n (Total N = 573)	(% of total)
**CTA/** **CTV**		
Positive CTA with positive CTV	74	(12.9%)
Positive CTA with negative CTV	52	(9.1%)
Negative CTA with positive CTV	30	(5.2%)
Negative CTA with negative CTV	417	(72.8%)
**Incidentaloma**	93	(16.2%)

*CTA* = Computed tomography angiography.

*CTV* = Computed tomography venography.

**Figure 1:**
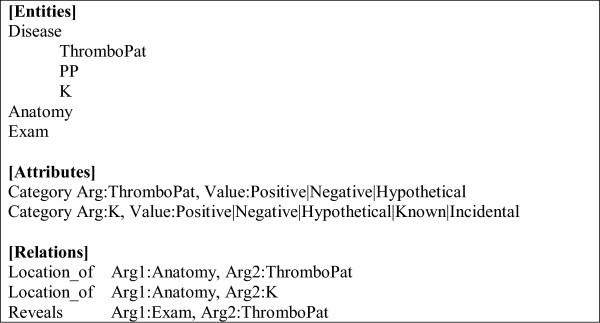
**The annotation scheme used in our project.**

We designed a knowledge representation scheme (Figure 1) that could be transposed to an annotation scheme modeling the diagnosis using three types of concepts: medical conditions (subdivided into thromboembolic diseases (“*ThromboPat*”), clinically relevant findings (“*K*”) and post-partum status (“*PP*”) ), affected body location (“*Anatomy*”) and diagnostic procedure (“*Exam*”). Thromboembolic diseases could be qualified with three modalities: positive, negative, hypothetical. Clinically relevant findings could also be qualified with two additional modalities: previously known and incidental. Finally, we considered relations between the medical conditions and affected anatomy parts (“*Location*_*of*”) and relations between exams and medical conditions (“*Reveals*”).

Lexicons for each concept type were compiled to help with the annotation task, with the following steps:

1. Identification of core concepts: an initial set of terms related to thromboembolic disease and its related anatomy location was obtained by pooling French MeSH terms from the Anatomy category, available synonyms [28] and the French translation of the Foundational Model of Anatomy [29].
2. Lexicon enrichment: As the terms in literature-oriented terminologies often did not reflect the way the same concepts were referred to in clinical narratives, we needed to add clinically-oriented term to the lexicon. Manual additions from a web-based thesaurus of terms linked to ICD-10 GM codes called Medcode [30] were added to our lexicon. Terms from Medcode were highly representative of the narratives of our radiology reports, listing abbreviations of terms, acronyms and narratives dialectal expressions of anatomy and thromboembolic disease.
3. Identification of clinically relevant finding terms within the reports required the help of a radiologist (DY). 93 radiology reports with incidental clinically relevant finding were identified. Terms that recalled clinically relevant findings such as “nodule” (nodule), “masse” (mass), or “adénopathie” (adenopathy) were added to our lexicon which compiled a total of 207 terms.

The lexicon comprised a total of 1242 terms, including 674 terms for anatomy, 278 for thrombopathology, and 207 for incidental findings.

Based on a recent survey of annotation tools for the biomedical domain [31], the Brat [32] interface was found to support annotation of entities, relationships and modalities, and allow the use of pre-annotations. After applying a simple lexicon matcher for concept recognition, annotations of the corpus were revised manually through the Brat interface.

Two annotation strategies were considered: “full annotation” and “light annotation”. The full annotation strategy consisted in thoroughly revising the concept pre-annotation on the entire reports (i.e. adding or removing concepts where the lexicon matcher failed, modifying boundaries for some concept annotations in order to produce as specific an annotation as possible), as well as creating modality and relation annotations on the entire reports. The light annotation strategy consisted in reasonably revising the concept pre-annotation (i.e. adding or removing concepts where the lexicon matcher failed), as well as creating modality and relation annotations. For thromboembolic diseases the annotation task focused on the conclusion section with attention given to the results section only if conclusion was incomplete. A conclusion was considered incomplete when it did not contain important findings described in other sections of the report. Typically, the conclusion section re-iterated the important findings of the exam (described in the result section) with respect to the indication. Incidental findings (which are unrelated to the indication) were often stated in the results section, but not in the conclusion. For clinically relevant findings, the annotation covered the entire report.

**Figure 2:**
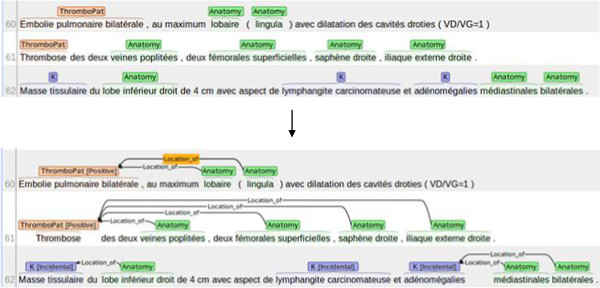
**Sample annotated text using Brat.** Top: Pre-annotated text using the automatic lexicon matcher. Bottom: Final annotations produced by the physician after revising the pre-annotations.

A computational linguist with extensive annotation experience (AN) annotated two sets of 10 radiology reports using the two strategies and calculated the annotation time. For the “full annotation”, the average annotation time was 20 minutes per report. The “light annotation” reduced the annotation time to 7 minutes per report. Due to time constraints, light annotation was performed on the entire corpus by a physician (ADP). Figure 2 shows a sample text with concept pre-annotations automatically produced by the lexicon matcher and the final annotations produced by the physician. Inter-annotator agreement (IAA) was then computed on the ten reports that were processed in common by the two annotators.

### Classification of radiology reports

**Figure 3:**
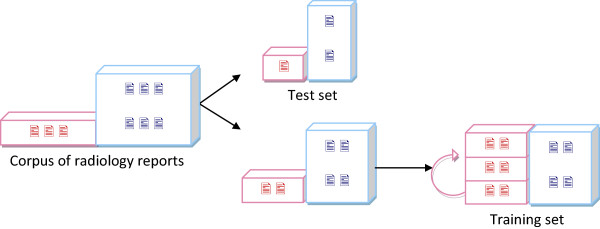
**Test and training set for CTA/CTV classification.**

At the HEGP University Hospital, the estimated prevalence for PE was around 24.5% and 15% for DVT [9], all patients with suspected embolism considered. Because of this, our extracted data set was highly imbalanced, with many more reports of negative CTA/CTV than positive CTA/CTV. To prevent our statistical model from over-predicting negative cases, we multiplied the number of positive cases and adjusted it to the number of negative cases. A 10-fold cross-validation to train the classifier could not be used as copies of a same positive case having a non negligible probability to be in the training set as well as in the test set, thus biasing the results. Therefore, we designed a test and a training set as follows: we first randomly selected 100 reports from our initial data set to form the test set. With the remaining set, we tripled the positive reports (in red on Figure 3), and adjusted the number of negative reports (in blue on Figure 3) to the number of positive reports. The test set remained imbalanced in order to reflect the reality of the data in the hospital. For the incidental findings classification, the positive cases from the training set were multiplied by 5. We validated this choice by testing other balancing parameters (replicating positive instances up to six times) and obtained similar results.

To perform the automatic classification of our radiology reports according to diagnosis, we used open-source tools: the Waikato Environment for Knowledge Analysis (WEKA) tool [33], and Wapiti [34, 35]. Our data was formatted into an attribute-relation file format (ARFF) using perl scripts. Initially we used a Weka Naïve Bayes classifier to test several feature sets and validate the usefulness of the annotations as features. Using binary encoding, the most useful features were: baseline plain text (text tokens), plain text with the adjunction of annotations, and finally text segmented by report section with the adjunction of annotations. Other features that we tested were: text tokens with filtering of unigram, filtering of uni- and bigrams, filtering of frequent and non-discriminating unigram stopwords, filtering of frequent and non-discriminating uni- and bigrams stopwords. In one model, we incorporated report section information by typing text and annotation features according to the section of the report they were extracted from. In another model, we only included text and annotation features coming from selected report sections.

Once the optimal feature sets were identified using the Naïve Bayes classifier, we developed an optimized classifier, using Wapiti implementations of Support Vector Machine (SVM) and Maximum Entropy (MaxEnt) algorithms.

### Evaluation

We evaluated our classification models using precision, recall, and F-measure. Precision was computed as the number of documents correctly classified as reporting a positive diagnosis over the total number of documents classified as reporting a positive diagnosis. Recall was computed as the number of documents correctly classified as reporting a positive diagnosis over the total number of documents reporting a positive diagnosis according to the gold standard. The F-measure was computed as the harmonic average between precision and recall.

IAA was used as a quality measure for the knowledge representation and the annotation task. It was computed as F-measure, which was shown to be equivalent to Cohen’s kappa for our type of annotation task [36]. IAA was obtained using the NICTA tool, an open-source tool made available by the National Information and Communication Technology Research Center of Australia [37].

## Results

### Annotated corpus

Table 1 presents the distribution of the thromboembolic diagnosis, exams used to make the diagnosis and incidental findings. Table 2 presents the results of the “light annotation” of the entire corpus for concepts, modalities and relations. Table 3 shows the inter-annotator agreement between the computational linguist and the physician on the subset of 10 radiology reports that were doubly-annotated. The overall inter-annotator agreement for entities was 77.3 (exact match) and 87.8 (inexact match), and for relations, 62.4 (exact match) and 71.8 (inexact match). Agreement is shown for the subset of relations that occurred in this small subset (for instance, there were no *Reveals* relations).

**Table 2:** **Distribution of annotations in the 573 radiology reports**

Concepts	N	Relations	N	Modalities	N
*Anatomy*	9702	*Location*_*of Reveals*	2507	*Negative*	1739
ThromboPat*	3116	*Reveals*	42	*Positive*	1653
*Exam*	1582			*Known*	293
K*	1478			*Incidental*	123
PP*	3			*Hypothetical*	118

*ThromboPat for thrombopathology concepts.

*K for clinically relevant findings.

*PP for post-partum.

**Table 3:** **Inter-annotator agreement on a sample of 10 radiology reports (F-measure)**

Category	Exact match	Inexact match
**Entities** (overall IAA)	77.3	87.8
*Anatomy*	73.5	90.9
ThromboPat*	95.7	99.1
*Exam*	89.3	89.3
K*	78.4	78.4
**Relations** (overall IAA)	62.4	71.8
*Anatomy Location*_of K*	80	80
*Anatomy Location*_of ThromboPat*	50	66.7

*IAA* = inter-annotator agreement.

*ThromboPat for thrombopathology concepts.

*K for clinically relevant findings.

### Automatic classification of diagnosis and findings

The performances of the Naïve Bayes and MaxEnt classifiers were evaluated on the 100 reports of the test sets (See Table 4). First, we used the Naïve Bayes classifier on a baseline model relying on plain text features and an enriched model using plain text and annotations for concepts, modality and relations. Once the optimal feature sets were identified using the Naïve Bayes classifier, SVM and MaxEnt algorithms were applied. We obtained the highest precision (1.00) and recall (0.95) for the identification of pulmonary embolism, using the MaxEnt classifier. Precision was 1.00 and recall was 1.00 for DVT. As for incidental findings, precision ranged from 0.32 (baseline) to 0.67 (baseline + annotations), and recall ranged from 0.29 (baseline) to 0.75 (sections + annotations). Results obtained with the SVM classifier were similar to the MaxEnt classification but slightly inferior (data not shown). Overall results for the MaxEnt classifier showed great improvements over the Naïve Bayes. Nonetheless result trends were similar to what we obtained with the Naïve Bayes classifier: using annotations improved the performance over the baseline in all cases.

**Table 4:** **Performances of the Naïve Bayes versus the maximum entropy classifiers on the CTA/CTV and incidentaloma test sets**

		Precision		Recall		F-measure	
Features		NB	ME	NB	ME	NB	ME
Baseline (plain text)	PE	0.78	0.88	0.96	0.95	0.86	0.91
	DVT	0.42	0.84	0.89	0.89	0.57	0.86
	PE and/or DVT	0.77	0.90	0.85	0.96	0.81	0.93
	Incidentaloma	0.32	0.43	0.29	0.32	0.30	0.37
Baseline + annotations	PE	0.99	1.00	0.97	0.95	0.98	0.98
	DVT	0.73	1.00	0.89	1.00	0.80	1.00
	PE and/or DVT	0.92	1.00	0.85	0.96	0.89	0.98
	Incidentaloma	0.67	NC	0.50	NC	0.57	NC
Baseline + annotations + section typing	Incidentaloma	0.46	NC	0.63	NC	0.53	NC
Critical sections* + annotations	Incidentaloma	0.60	0.76	0.75	0.81	0.67	0.80

*NB*: Naïve Bayes; *ME*: Maximum Entropy.

*PE*: Pulmonary Embolism; *DVT*: Deep Vein Thrombosis.

*results and conclusion sections.

*NC*: Not Calculated.

## Discussion

### Contribution of automated analysis of radiography reports to the characterization of patients admitted for PE

CTA and CTV are routinely used at HEGP to evaluate patients with suspicion of PE. This manuscript reports on the very good performance of an automated approach to determine the thrombotic status from unstructured CT reports. This approach may be used in many applications, such as eligibility screening in clinical trials or analysis of retrospective e-cohorts.

This study showed that in 30 reports out of 573 a diagnosis of thromboembolic disease was reached with CTV while CTA did not show any evidence of thromboembolism. Similarly, CTA helped reach a diagnosis of DVT in 52 cases while CTV was negative (see Table 1). These results suggest that the two techniques are complementary. The classification method used in this study has the potential to help clinicians extract the reports providing crucial information on diagnosis yield and evaluate the contribution of each technique.

CT exams have the ability to detect unexpected non-vascular findings, with clinical, ethical and financial implications of incidental imaging findings. A significant proportion of these findings may be serious and need to be adequately managed. An increasing amount of attention has been paid to incidental findings, e.g., the ACR Incidental Findings Committee II recently published four papers, based on repeated reviews and revisions and a collective review and interpretation of relevant literature [38]. In this context, the automated analysis of patient records provides a valuable data driven approach to characterize the incidence and prevalence of such findings (16.2% in our study, Table 1), describe the lesions most frequently seen on CTs performed on patients admitted for suspicion of thromboembolism, and develop protocols for follow-up and treatment.

### Contribution of knowledge representation and NLP to the analysis of radiography reports

The annotation of a large corpus of CTA/CTV reports showed that relations between disease concepts and anatomy concepts are explicitly stated in the reports (according to Table 2, there are 2,507 “*Location*_*of*” relations in the 573 reports in our corpus, an average of almost 5 relations per report). This means that automatically extracting these relations using NLP can provide very specific information about the anatomic site of the thrombus. In contrast, in most cases, the relations between exams and diseases are implicit: there were only 42 “*Reveals*” relations between exams and diseases in the study corpus. Interestingly, when the “*Reveals*” relations are removed from the feature set in the best models, there is no difference in performance (data not shown). The modality annotations (see Table 2) show that a high number of concepts are assigned a negative (N = 1,739) or hypothetical (N = 118) modality, compared to a positive modality (N = 1,653). This highlights the need for an analysis that goes beyond concept extraction [39]. In this study, we established an annotation schema as a method to provide both medical and linguistic knowledge representation to model diagnosis [40, 41]. Machine learning using a simple Naïve Bayes classifier was able to automatically detect thromboembolic diagnosis with high precision and recall (F-measure above 0.80 for the best models, according to Table 4). A more advanced classifier obtained even better results (See Maximum Entropy results in Table 4) with F-measure above 0.98. The use of concepts, modalities and relations as features significantly improved the results, especially for DVT where F-measure increased from 0.57 to 0.80 (40% improvement) in the Naïve Bayes model.

Identifying incidentaloma initially proved more difficult (best F-measure was 0.67 using Naïve Bayes, per Table 4) but the use of concepts, modalities and relations still contributed to increase F-measure from 0.30 to 0.57 (90% improvement). The Maximum Entropy classifier provided results on par with thromboembolic diagnosis classification (F-measure of 0.80 as shown in Table 4). It is important to point out that there are no specialized lexicons for incidental findings. Imaging signs are not represented in established terminologies such as MeSH. Shore *et al*. incorporated a total of 1,135 terms for imaging signs into the RSNA’s RadLex ontology of radiology terms [42]. This ontology is available for English and German, but not yet for French. A contribution of the corpus annotation performed in this study is that it allowed us to gather some of these terms. A comprehensive set of terms for imaging signs should help to normalize clinical reporting and indexing and improve the power of NLP tools to understand the content of narrative radiology reports.

### Comparison to other work

A similar study which aimed to identify exams ordered to rule out PE and then access and review the characteristics of the resulting exam was conducted by Chapman *et al*. [39]. They developed a text processing application using pyContext annotations to perform rule-based classification of CTA reports in English and achieved a precision of 0.83 and a recall of 0.98 for pulmonary embolism, which is comparable with our recall of 0.95 but less than our precision of 1.00.

Along with the context of incidentaloma, two studies addressed the automatic identification of clinically important follow-up recommendations from radiology reports in English [43, 44]. Yetisgen-Yildiz *et al*. [43] used a MaxEnt algorithm and measured the impact of data imbalance between positive and negative classes and several feature types: unigram, Ngram, syntactic, knowledge-based, and finally structural features. The latter highlighted the potential importance of section header information in the classification decision. In our model, we also focused our classification processing on specific sections. Their best achieved F-score was 0.758, with precision of 0.662 and recall of 0.885. Dutta *et al*. [44] undertook a prospective study on automatically identifying incidental clinically important follow-up recommendations on a larger corpus (3,000 reports, covering various specialties) using an NLP algorithm based on keywords.

### Limitations of this study

A limitation of this study lies with the corpus selection. The refined query used to perform the final corpus selection only achieves 61% recall on a test sample of 200 documents. While the content of the relevant reports that were not selected (false negatives) did not look different from those that were selected, a bias may remain. It can also be noted that the proportion of documents selected by the query on the test sample (78/200) is higher than overall (573/6758). However, overall, the refined query achieved our goal of retrieving 100% of reports corresponding to CTA and CTV prescribed in the context of PE. Furthermore, the distribution of reports in the corpus was 22% PE, and 18% DVT, which did not differ significantly from the expected distribution from the literature, respectively 24.5% and 15% [9]. In future work, we will be able to leverage the whole set of EHR data (e.g. procedure codes which were are not available at the time of our study) from the data warehouse.

Due to time constraints, we performed a “light annotation” of the 573 health records in the study corpus. While these “light” annotations proved to be useful features for the automatic classification of the radiology reports, in order to fully automate the classification process, we need to generate the annotations for concepts, modalities and relations automatically. The annotated corpus produced in this study is a valuable resource that we will use in future work for developing appropriate statistical annotation models. Error analysis will also be performed on a larger and richer data set.

## Conclusions

Electronic health records are becoming increasingly prevalent in hospitals. As much of the clinical data is available in free-text format, processing clinical narratives has become a major challenge and an enabling technology for improving clinical research and patient care. Our project shows that NLP can be useful to carry out a large-scale retrospective study in the field of thromboembolic diseases. Our project provides automatic classification of reports that is not possible to obtain from the coding of patient records. In future work, we want to develop an end-to-end automatic system that can automatically annotate the reports as well as perform classification. We also plan to extend our project on incidental findings. Indeed, the identification of potentially serious incidental finding is by no means limited to CTA in the context of PE. Developing a text processing pipeline for identifying incidental findings for epidemiological surveillance would help clinicians clinically examine their use of new imaging techniques.

## Abbreviations

NLP: Natural language processing
CT: Computed tomography
CTA: Computed tomography angiography
CTV: Computed tomography venography
PE: Pulmonary embolism
DVT: Deep vein thrombosis
HEGP: Hôpital Européen Georges Pompidou
IAA: Inter-annotator agreement.
